# Coincidence of Tethered Cord, Filum Terminale Lipoma, and Sacral Dural Arteriovenous Fistula: Report of Two Cases and a Literature Review

**DOI:** 10.3389/fneur.2018.00807

**Published:** 2018-09-27

**Authors:** Łukasz Przepiórka, Przemysław Kunert, Paulina Juszyńska, Michał Zawadzki, Bogdan Ciszek, Mariusz Głowacki, Andrzej Marchel

**Affiliations:** ^1^Department of Neurosurgery, Medical University of Warsaw, Warsaw, Poland; ^2^Department of Radiology, Centre of Postgraduate Medical Education, Warsaw, Poland; ^3^Division of Interventional Neuroradiology, Department of Radiology, Central Clinical Hospital of Ministry of the Interior and Administration, Warsaw, Poland; ^4^Department of Descriptive and Clinical Anatomy, Medical University of Warsaw, Warsaw, Poland; ^5^Department of Neurosurgery, John Paul II Western Hospital, Grodzisk Mazowiecki, Poland

**Keywords:** spinal dural arteriovenous fistula, tethered cord syndrome, lipoma, surgery, embolization

## Abstract

Spinal dural arteriovenous fistula (SDAVF) is the most common vascular malformation of the spine in adults. However, the coincidence of tethered cord syndrome, lipoma, and SDAVF on the sacral level is exceptionally rare. We describe two patients, probably the fifth and sixth ever reported. The first was a 33 year-old female who underwent surgical cord de-tethering. Surprisingly, a sacral SDAVF was discovered intraoperatively, despite negative digital subtraction angiography (DSA). The second patient was a 30 year-old male with similar pathologies. After three failed embolizations, the fistula was surgically disconnected. Both patients recovered well. A review of patients with sacral SDAVF coexisting with spinal dysraphism, with an emphasis on the basis of symptoms was done. As a rule, in these coincident disorders, the SDAVF was the direct cause of increasing symptoms. Previous reports and our findings reveal that surgery might be superior to endovascular embolization for treating sacral SDAVFs with coexisting entities, because surgery offers a one-step treatment.

## Introduction

Tethered cord syndrome (TCS) is a developmental neurological disorder caused by an abnormally low conus medullaris. It typically presents in childhood, but it may persist undetected until adulthood, and it may be associated with an intradural lipoma. Patients with coexisting abnormalities (e.g., myelomeningocele) and clear clinical symptoms (e.g., progressive leg weakness, urinary incontinence) may require surgical treatment. The goal of surgery is to de-tether the spinal cord to relieve spinal cord stretching. Nevertheless, the indications for surgery remain controversial (1).

In contrast, the spinal dural arteriovenous fistula (SDAVF) is an acquired condition. It is the most common vascular malformation in the spine in adults. It is formed in the neural foramen by an abnormal, direct connection between the dural artery (which supplies the dural root sleeve and adjacent spinal dura) and the medullary vein (which drains the coronal venous plexus) (2, 3).

It may present with back pain and progressive neurological deficit. A SDAVF typically requires either surgical or endovascular management.

The coincidence of TCS, lipoma (or lipomyelomeningocele), and an SDAVF at the sacral level is very rare. In this communication we report two such patients. (4–6).

## Case material

### Case 1

**Figure 1:**
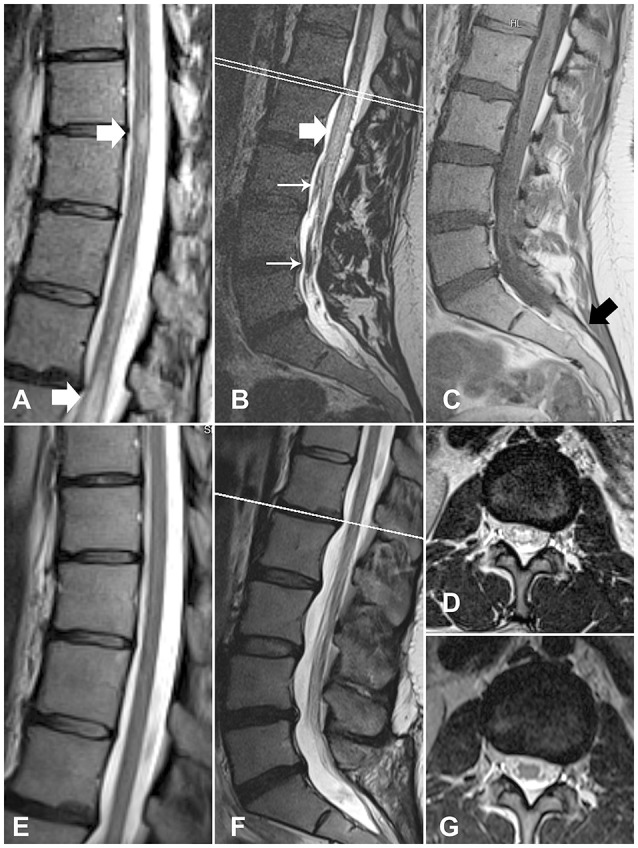
MR images of the spinal cord of case 1, before and after treatment. **(A)** Myelopathic changes in the thoracic spinal cord (white arrows) (**B)** Myelopathic changes in the conus medullaris (thick arrow) and thickened torturous vessels (thin arrows). **(C)**T1WI sequence shows a filum terminale lipoma (black arrow). (**D)** A transverse section at the L1/L2 level shows myelopathic changes. (**E–G)** Follow-up images demonstrate the resolution of myelopathic changes and the disappearance of the thickened veins.

A 30-year-old female presented with a 10-year history of pain in the lumbosacral spine; she had had casual radiation to both lower limbs. After her first delivery, she developed back pain. The MRI demonstrated a tethered cord at the L4 level and a filum terminale lipoma. The MRI also showed tortuous veins on the spinal cord surface (Figure 1C). Nine years later, during the second pregnancy, she noted weakness and sensory loss, imbalance with urinary and fecal incontinence. On admission, she had bilateral plantar flexion weakness (grade 4 according to modified Medical Research Council system) and reduced sensation in the gluteal regions and legs, and plantar response. The Babinski and Rossolimo signs were present bilaterally. A repeat MRI revealed intraspinal T2 hyperintensive changes in the thoracic spine and conus (Figures 1A,B,D). Those changes were consistent with neurologic deficit and, after exclusion of inflammatory demyelinating diseases, based on a brain MRI and an aquaporin-4 antibody test, diagnosed as myelopathy. Due to described torturous veins on the spinal cord surface (Figure 1B), the patient underwent spinal digital subtraction angiography (DSA). The range of DSA was from the Th6 level to the coccygeal artery. The results did not reveal any vascular malformation. Therefore, the preliminary diagnosis was symptomatic TCS and filum terminale lipoma. In view of weakness and neurological deficit surgical spinal cord de-tethering, without lipoma resection was planned.

The sacral canal was opened with a median incision. The dura was thin and transparent. After a midline dura and arachnoid incision, a tumor was visualized that appeared to be a lipoma. It engulfed the filum terminale and spinal nerve roots caudally, to the S2 level. The S1 and S2 nerve roots were positioned lateral to the tumor. A thickened, tortuous, bright-red vessel was also noted on the filum terminale, running in the cranial direction. The external surface of the dura was explored bilaterally at the sacral level in search for a SDAVF. A clearly visible nidus was identified at the S3 level on the left side. An empirical test was performed to support this finding. A clip was placed on the arterialized vein, intradurally, for a few minutes. Then, the vessel on the filum terminale clearly changed color, from bright red into bluish gray, and its tension decreased. The SDAVF was then intradurally disconnected, and the nidus was extradurally coagulated. In addition, the filum terminale was cut, and samples of lipoma were taken for histology. The dura was sutured in a watertight fashion, and the wound was closed in layers.

The postoperative course was uneventful. A few days later, the patient was discharged. A follow-up MRI showed resolution of the myelopathic changes (Figures 1E–G). At the 1-year follow-up, the patient had considerably improved, with no incontinence or motor deficit.

### Case 2

**Figure 2:**
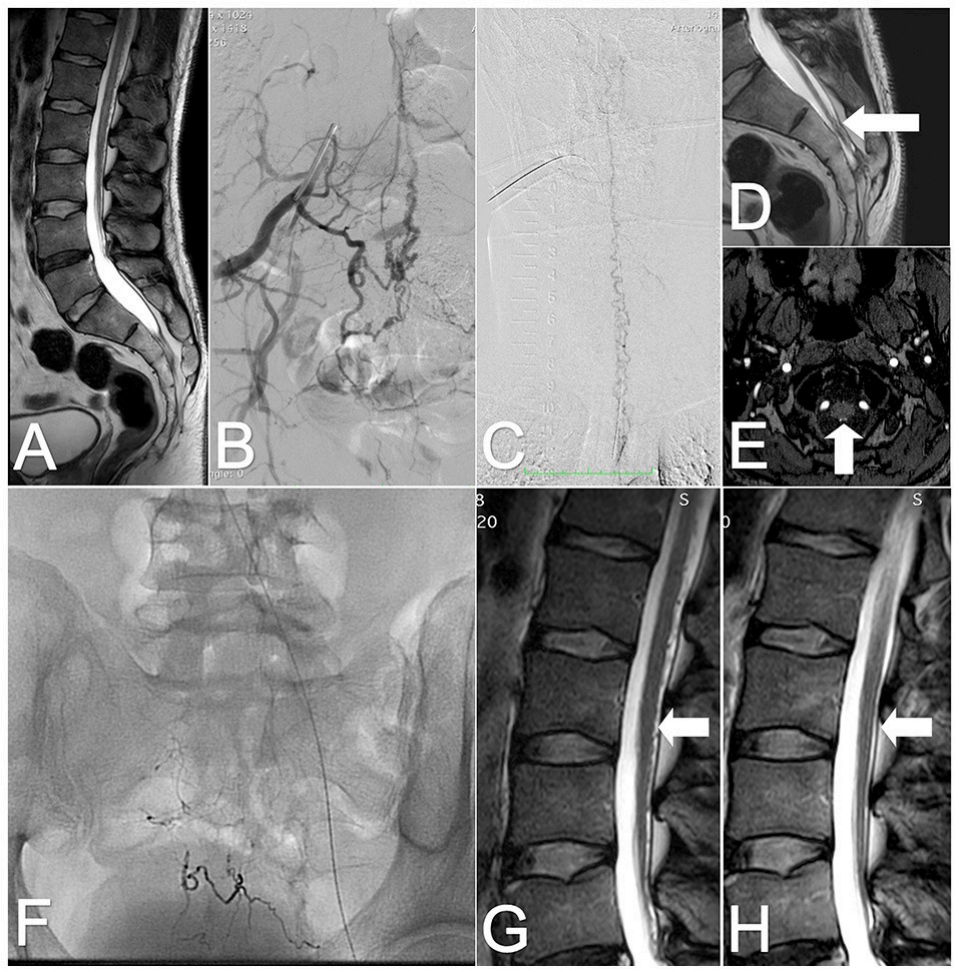
Images of the spinal cord of case 2, before and after treatment. (**A)** T2-weighted MRI: Tethered cord and sacral lipoma. **(B)** DSA: SDAVF at the S2-S3 level supplied by the lateral sacral arteries. **(C)** DSA: Tortuous veins on the posterior surface of the cervical spinal cord. **(D)** T2-weighted MRI: Tortuous vein on the posterior surface of the tethered spinal cord (arrow). **(E)** TOF MRI: Draining vein (arrow) at the atlanto–occipital junction level. **(F)** X-ray of the sacral area shows embolic agents after three embolizations. **(G)** Lumbar T2-weighted MRI: After the last embolization, tortuous vessels are visible on the posterior surface of the spinal cord (arrow). **(H)** Follow-up MRI: at 6 months after the surgery, the tortuous veins are no longer observed on the posterior surface of the spinal cord (arrow).

A 33-year-old male presented with a 2-year history of pain in the lumbosacral spine with progressive lower-limb weakness, urinary frequency, and incontinence. Symptoms intensified after exercise and prolonged standing. Lumbar spine MRI demonstrated a tethered cord at the L3 level, a filum terminale lipoma, and edema in the spinal cord. It also showed tortuous veins on the posterior surface of the spinal cord (Figures 2A,D).

The neurological examination revealed bilateral hip-joint flexion weakness (grade 4 according to modified Medical Research Council system), plantar flexion weakness (grade 3 according to modified Medical Research Council system), and reduced sensation in the S2–S3 dermatomes. The Babinski and Rossolimo signs were present bilaterally. Based on the clinical and radiological signs, a SDAVF was suspected. A DSA confirmed the SDAVF at the S2-S3 level, in the dura supplied by lateral sacral arteries and branches from the internal iliac arteries. Tortuous draining veins were present on the posterior surface of the entire spinal cord and terminated intracranially (Figures 2B,C,E).

The patient underwent three endovascular embolizations, each with a different agent. First, embolization was performed with Onyx™ 18, administered in two microcatheters. This treatment resulted in an occlusion of the fistula. The patient significantly improved, but due to poor penetration of the draining vein, after a few weeks, the fistula recanalized and symptoms returned.

A second embolization was performed 4 months later. The vascular access to the fistula was more complicated. A 25% Phil™ injection was feasible only through the small sacral branches of the left internal iliac artery. This procedure achieved a significant reduction of flow through the fistula. Eighteen months later embolization of the fistula was attempted with diluted glue. Despite good occlusion of the fistula observed in a DSA, the tortuous vessels remained visible in the follow-up MRI (Figures 2F,G). The symptoms persisted, and the patient was qualified for surgical treatment.

The goal of surgery was to occlude the draining vein and the arterial feeder and to reduce the lipoma. With a median incision, the sacral canal was opened on the right side. The dura was thick, and there were multiple vessels filled with embolic agents. There was one large, bright-red vessel extradurally and one intradurally at the S2-S3 level. A clip was placed upon the arterialized vein intradurally for a couple of minutes. After that, the vessel changed color and the tension decreased. Both intra- and extradural vessels were coagulated. Afterwards, the lipoma was visualized and partially removed. The dura was sutured in a watertight fashion, and the wound was closed in layers.

The postoperative course was uneventful, and a few days later, the patient was discharged. After 6 months, a follow up MRI showed the absence of tortuous vessels on the posterior surface of the spinal cord and a significant reduction in the myelopathic changes (Figure 2H). On follow-up, the patient considerably improved, with no incontinence, some urinary frequency and mild weakness (grade 4 according to modified Medical Research Council system).

## Discussion

Co-occurrence of TCS, an intradural lipoma, and a SDAVF at the sacral level, although exceptional, should be kept in mind, in light of the growing number of reported cases. This study described the fifth and sixth such cases, with a TCS and a SDAVF at the sacral level (Table 1). Recently, one of these cases was listed by Horiuchi et al. and two more were added by Talenti et al. (6, 8).

**Table 1:** Literature review on the coincidence of sacral dural arteriovenous fistula (DAVF), tethered cord syndrome (TCS), and filum terminale lipoma/lipomyelomeningocele.

**Author, year**	**Sex, age (y)**	**Vascular malformation description**	**Lipoma, lipomyelomeningocele, other spinal dysraphism**	**Clinical presentation**	**Duration of symptoms**	**Description of previous surgery**	**Treatment**	**Outcome**
Djindjian et al. (5)	M, 53	Sacral DAVF supplied by lateral sacral artery	Filium terminale lipoma	Paraparesis	1 year	0	Lipoma: complete resection; AVF: resection	Unchanged at 6 -month follow-up
Krisht et al. (4)	F, 58	DAVF with arterial feeders from the bilateral lateral sacral arteries and right internal iliac artery	Lumbar LMC	Paraparesis, sensory loss, and bladder urgency	2 years	L4/L5 laminectomy for de-tethering in another department	Lipoma: resection; AVF: Onyx injection (incomplete occlusion) and subsequent surgical resection	After 6 months, almost complete regain of motor function
Talenti et al. (6)	M, 19	L5-level fistula supplied by a sacral branch from the right hypogastric artery; this SDAVF, at S1–S3 level, consisted of two fistula points with arterial feeders	Lumbosacral LMC, cloacal exstrophy	Severe paraparesis, bilateral club foot, and slight bilateral triceps surae retraction	1 year	At age 6, partial resection of the lipomyelomeningocele; at age 7, complex pelvic surgery with bladder and anal reconstruction	Laminectomy, then, after discovering the fistulas, embolization of both	After 4 months, was able to walk using crutches; sensitivity, bowel control, and urinary control were preserved
Talenti et al. (6)	F, 53	DAVF at S1 level, fed by a right sacral segmental branch and draining into ectatic perimedullary veins	Sacral lipomyelocele	No bladder control, pathological patellar reflexes, spastic paraparesis, and dysesthesias	8 months	Surgery at 1 year of age for detachment of a sacral meningocele, followed by 2 other lumbar surgeries	Onyx, followed by a partial resection of the lipoma and coagulation of the fistula	Persistent spastic paraparesis and neurologic bladder that required self-catheterization
Przepiórka et al. (7)	F, 30	Sacral SDAVF discovered intraoperatively	Filium terminale lipoma	Progressive paraparesis, urinary incontinence.	10 years	0	SDAVF: disconnected, coagulated	All deficits resolved at the 1 year follow-up
Przepiórka et al. (7)	M, 33	SDAVF at the S2-S3 level supplied by lateral sacral arteries and branches from internal iliac arteries	Filium terminale lipoma	Bilateral hip joint flexion weakness, plantar flexion weakness, pathological signs present bilaterally	2 years	0	3 failed embolizations (Onyx, Phil, diluted glue); successful surgery, coagulation of the malformation, partial resection of the lipoma	Considerable improvement: no incontinence, some urinary frequency, and reduced motor deficit

*AVF, arteriovenous fistula; DAVF, dural AVF; LMC, lipomyelmeningocele; SDAVF, sacral DAVF; TCS, tethered cord syndrome*.

The cause of this triple coincidence is enigmatic. On one hand, the SDAVF is believed to be an acquired entity, in contrast to primary spinal anomalies. This feature suggested that the coexistence of these pathologies might be accidental. On the other hand, lipomas, and angiomas both arise from a mesenchymal origin. Previous reports of combined intramedullary AVM and lipoma suggest that there may be a correlation between these etiologies (9, 10). However, to the best of our knowledge, no previous studies have described the coexistence of dysraphic anomalies and a SDAVF at the sacral level, which presented in childhood. Also, a Medline database search did not reveal any report on a congenital SDAVF. However, several reports have described a SDAVF secondary to either surgery or trauma (11–15). Therefore, theoretically, a previous surgery for a myelomeningocele could subsequently trigger the formation of a SDAVF.

Previous studies have reported that a venous sinus thrombosis with associated intravenous hypertension and a venous outflow obstruction might initiate the formation of shunts (16–18). Therefore, an alternative explanation of the coexistence of disorders found in our cases might have been that a lipoma caused pressure, which obstructed the venous outflow, and thus, promoted the formation of a SDAVF. This alternative mechanism would be unrelated to a previous history of trauma or surgery.

Acquired SDAVFs are typically located in the thoracolumbar region, and the mean age at presentation is around 60 years (19). Sacral SDAVFs are rare, and the mean age of presentation of SDAVFs that coexist with a TCS is 41 years (Table 1). Therefore, it is also possible that a congenital anomaly, such as an intradural lipoma, might promote the formation of a SDAVF at a younger age. Recently, Horiuchi et al. described two cases and reviewed nine previously published cases of SDAVFs and AVMs that coexisted with spinal lipomas of different types and at different spinal levels (8). Both Djindjian et al. and Horiuchi et al. postulated that the most probable mechanism underlying a delayed SDAVF formation might be adipocyte release of angiogenic factors; these factors can influence angiogenesis (20) and lead to local hypervascularization (5, 8). Moreover, Jellema et al. excluded prothrombotic factors in the pathogenesis of SDAVFs (21).

The symptomatic coincidence of TCS, sacral lipoma, and SDAVF gives rise to the question: what exactly drives the symptoms? Talenti et al. recently described diagnostic and therapeutic difficulties for two similar cases (6). In both those patients, an initial de-tethering of the spinal cord and removal of the lipoma/lipomyelomeningocele did not result in improvement or deterioration. Following spinal angiography, they next addressed the spinal vascular pathology. The natural history of the filum terminale lipoma is benign; only 5% of patients present with TCS symptoms (22). Thus, the lipoma was the least likely cause of neurological impairment. Nonetheless, to some extent, both TCS and SDAVF could cause similar symptoms. A TCS becomes symptomatic more frequently in children than in adults (23). In contrast, impairments related to SDAVFs develop in older and middle-aged individuals. Moreover, sacral SDAVFs induce spinal cord hyperintensities, visible in T2-weighted MRIs, which typically disappear after successful treatment (24). Therefore, the most probable cause of neurological deterioration is the SDAVF, based on the average age of patients with coincidental TCS, sacral lipoma, and SDAVF (see Table 1) and based on the good postoperative outcome; i.e., the resolution of intramedullary changes, demonstrated in the MRIs. Furthermore, unlike TCS, SDAVF symptoms can become exacerbated with physical effort and with increasing intra-abdominal pressure, as in both cases described in the present study.

In patients with this triple coincidence, the SDAVF was always the direct cause of increasing symptoms. Therefore, the main goal of treatment should be occlusion. In the literature, endovascular embolization appeared to be equally as effective as surgery, and embolization was the sole treatment in similar cases (24). Unfortunately, embolization was either unfeasible or ineffective in our cases. With surgery, both the SDAVF and TCS could be explored and treated in one procedure.

In our first case, the initial goal of surgery was to de-tether the spinal cord, because the SDAVF was not confirmed on a preoperative DSA. However, the question remains, why was the SDAVF obscured on the DSA? Oldfield et al. described three cases of SDAVFs that were undetected in a DSA, but they were highly suspected, based on other neuroimaging modalities. Those SDAVFs were successfully disconnected during surgery (25). The factors that impeded spinal DSA detection included obesity, aortic aneurysm, aortic atherosclerosis with tortuosity, and orifice obstruction or tortuosity of the lumbar arteries. In our case, a potential explanation for the false negative DSA results could be that the DSA was not sufficiently thorough; thus, we did not examine the sacral level inflow.

The two cases described here showed that sacral SDAVFs may be difficult to detect in DSAs and hard to occlude with endovascular embolization. Previous studies presented in the Table 1 also described unsuccessful embolizations in treating SDAVFs (4, 6, 8). In total, there have been 5 unsuccessful attempts of endovascular occlusion in 3 out of 6 reported cases (Table 1). Thus, we concluded that a surgical approach might be superior to endovascular embolization, because surgery offers a one-step treatment.

## Concluding remarks

The co-occurrence of TCS, lipoma, and SDAVF at the sacral level is exceptionally rare. Previous reports and our findings reveal that surgery might be superior to endovascular embolization for treating sacral SDAVFs with coexisting entities, because surgery offers a one-step treatment. A growing number of reported cases have indicated that the symptoms of this triple disorder may be caused solely by the SDAVF, and not by the TCS or lipoma, which are clearly visible in neuroimaging.

## Consent

A written informed consent was obtained from the participants for the publication of this case report.

## Author contributions

PK designed the project and critically reviewed it. ŁP prepared the manuscript, collected and reviewed data. PJ, MZ, and MG collected case data. BC reviewed the anatomy. AM did final approval of the version to be published.

### Conflict of interest statement

The authors declare that the research was conducted in the absence of any commercial or financial relationships that could be construed as a potential conflict of interest.
